# 3-(6-Benz­yloxy-2,2-dimethyl­perhydro­furo[2,3-*d*][1,3]dioxolan-5-yl)-5-(4-chloro­phen­yl)-4-nitro-2-phenyl-2,3,4,5-tetra­hydro­isoxazole

**DOI:** 10.1107/S1600536809034096

**Published:** 2009-09-05

**Authors:** M. NizamMohideen, M. Damodiran, A. SubbiahPandi, P. T. Perumal

**Affiliations:** a Department of Physics, The New College (Autonomous), Chennai 600 014, India; b Organic Chemistry Division, Central Leather Research Institute, Chennai 600 020, India; c Department of Physics, Presidency College (Autonomous), Chennai 600 005, India

## Abstract

In the title compound, C_29_H_29_ClN_2_O_7_, the isoxazole and dioxolane rings adopt envelope conformations, and the furan ring adopts a twisted conformation. The crystal structure is stabilized by inter­molecular C—H⋯π inter­actions between a benz­yloxy methyl­ene H atom and the 4-chloro­phenyl ring of an adjacent mol­ecule, and by weak non-classical inter­molecular C—H⋯O hydrogen bonds. In addition, the crystal structure exhibits a Cl⋯O halogen bond of 3.111 (3) Å, with a nearly linear C—Cl⋯O angle of 160.7 (1)°.

## Related literature

For general background to 1,3-dipolar cyclo­addition reactions, see: Gothelf & Jorgensen (1998 ▶); Bernotas *et al.* (1996 ▶); Breuer (1982 ▶); Colombi *et al.* (1978 ▶); Hossain *et al.* (1993 ▶). For ring puckering parameters, see: Cremer & Pople (1975 ▶); Nardelli (1983 ▶). For halogen bonds, see: Politzer *et al.* (2007 ▶).
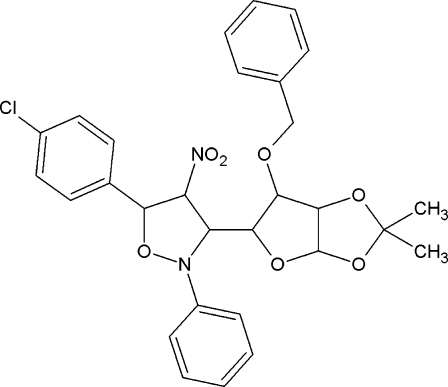


## Experimental

### 

#### Crystal data

C_29_H_29_ClN_2_O_7_
*M* *_r_* = 552.99
Orthorhombic, 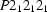

*a* = 12.7862 (5) Å
*b* = 13.0160 (5) Å
*c* = 16.8232 (6) Å
*V* = 2799.80 (18) Å^3^
*Z* = 4
Mo *K*α radiation
μ = 0.19 mm^−1^
*T* = 293 K
0.3 × 0.2 × 0.2 mm

#### Data collection

Bruker Kappa APEXII CCD diffractometer
Absorption correction: multi-scan (*SADABS*; Bruker 2004 ▶) *T*_min_ = 0.926, *T*_max_ = 0.964
17056 measured reflections
4764 independent reflections
3446 reflections with *I* > 2σ(*I*)
*R*_int_ = 0.034

#### Refinement

*R*[*F* ^2^ > 2σ(*F* ^2^)] = 0.037
*wR*(*F* ^2^) = 0.088
*S* = 1.02
4764 reflections
355 parameters
1 restraint
H-atom parameters constrained
Δρ_max_ = 0.15 e Å^−3^
Δρ_min_ = −0.14 e Å^−3^
Absolute structure: Flack (1983 ▶), 1975 Friedel pairs
Flack parameter: −0.05 (8)

### 

Data collection: *APEX2* (Bruker, 2004 ▶); cell refinement: *APEX2* and *SAINT* (Bruker, 2004 ▶); data reduction: *SAINT* and *XPREP* (Bruker, 2004 ▶); program(s) used to solve structure: *SHELXS97* (Sheldrick, 2008 ▶); program(s) used to refine structure: *SHELXL97* (Sheldrick, 2008 ▶); molecular graphics: *ORTEP-3* (Farrugia, 1997 ▶) and *DIAMOND* (Brandenburg, 1998 ▶); software used to prepare material for publication: *SHELXL97* and *PLATON* (Spek, 2009 ▶).

## Supplementary Material

Crystal structure: contains datablocks global, I. DOI: 10.1107/S1600536809034096/lx2103sup1.cif

Structure factors: contains datablocks I. DOI: 10.1107/S1600536809034096/lx2103Isup2.hkl

Additional supplementary materials:  crystallographic information; 3D view; checkCIF report

## Figures and Tables

**Table 1:** Hydrogen-bond geometry (Å, °)

*D*—H⋯*A*	*D*—H	H⋯*A*	*D*⋯*A*	*D*—H⋯*A*
C13—H13⋯O2^i^	0.98	2.54	3.298 (3)	134
C17—H17*B*⋯O1^ii^	0.97	2.46	3.218 (3)	135
C21—H21⋯*Cg*1^i^	0.93	2.75	3.598 (1)	152

Symmetry codes: (i) 
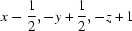
; (ii) 
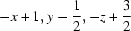
. *Cg*1 is the centroid of the C1–C6 ring.

**Fig. 1.**
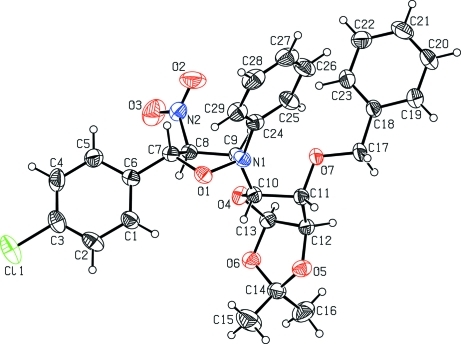
The molecular structure of the title compound with the atom numbering scheme. Displacement ellipsoids are drawn at the 50% probability level. H atoms are presented as a small spheres of arbitrary radius.

**Fig. 2.**
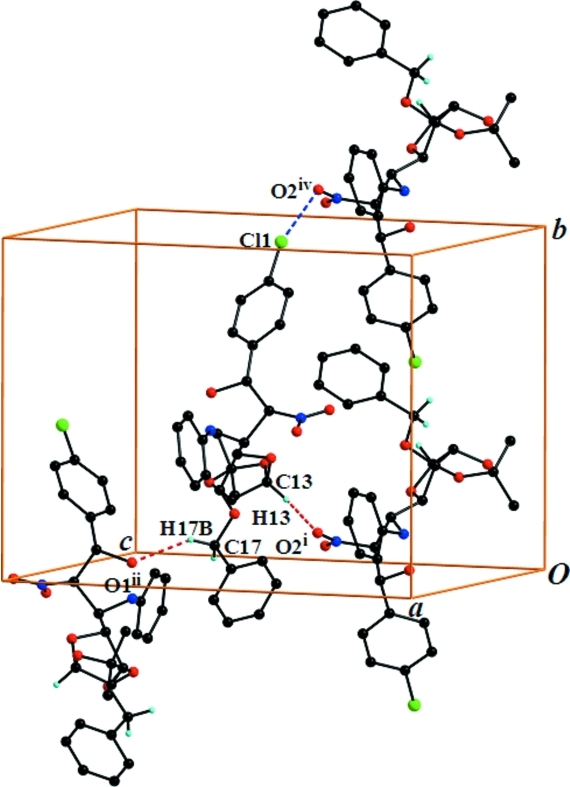
The C–H···O interactions, and Cl1···O halogen bond (dashed lines) in the title compound. [Symmetry codes: (i) x - 1/2, -y + 1/2, -z + 1; (ii) - x + 1, y - 1/2, -z + 3/2; (iv) x - 1/2, - y + 3/2, -z + 1.]

**Fig. 3.**
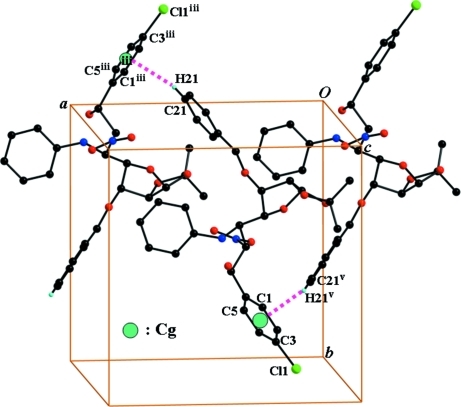
C–H···π interactions (dashed lines) in the title compound. Cg denotes the ring centroid. [Symmetry codes: (iii) x + 1/2, - y + 1/2, - z + 1; (v) x - 1/2, - y + 1/2, - z + 1.]

## supplementary crystallographic information

### Comment

The 1,3-dipolar cycloaddition of nitrones with olefinic dipolarophiles proceeds through a concerted mechanism yielding highly substituted isoxazolidines (Gothelf & Jorgensen, 1998). The cornerstone for cycloaddition reactions, nitrones, are excellent spin traping (Bernotas *et al.*, 1996) and highly versatile synthetic intermediates (Breuer, 1982). Highly substituted spiro-isoxazolidines result from the 1,3-dipolar cycloaddition of exocylic olefins with nitrones and these spiro-isoxazolidines have also been transformed into complex heterocycles (Colombi *et al.*, 1978, Hossain *et al.*, 1993). Here we report the crystal structure of the title compound (Fig. 1).

The dihedral angle between the phenyl rings C1-C6 and C18-C23, C18-C23 and C24-C29, and, C1-C6 and C24-C29 are 8.4 (2), 83.9 (1) and 75.7 (1)°, respectively. The five membered tetrahydrofuran ring (C10-C13/O4) adopts a twisted conformation and the other five membered rings, isoxazole ring (C7-C9/O1/N1) and the dioxolan ring (C12-C14/O5/O6), adopt envelope conformations on C11 and C10, O1 and O5 with a pseudo-twofold axis passing through C12–C11, C9–C8 and C13–O6 bonds. The puckering parameters (Cremer & Pople, 1975) and the lowest displacement asymmetry parameters (Nardelli, 1983)as follows: for the tetrahydrofuran ring q_2_ = 0.365 (1) Å, φ = 304.9 (1)°, Δ_S_ (C10) is 12.8 (1)° and Δ_2_ (C13) is 1.8 (1)°, for the isoxazolidine ring q_2_ = 0.377 (1) Å, φ = 359.7 (1)°, Δ_S_ (O1) is 1.0 (1)° and Δ_2_(C8) is 19.6 (1)° and for the dioxolone ring q_2_ = 0.248 (1) Å, φ = 181.2 (1)°, Δ_S_ (O5) is 0.3 (1)° and Δ_2_ (C13) is 13.3 (1)°.

The molecular packing is stabilized by weak non-classical intermolecular C–H···O hydrogen bonds (Table 1 and Fig. 2; symmetry code as in Fig. 2). Additionally, the crystal structure exhibits a Cl···O halogen bond (Politzer *et al.*,  2007) between the chlorine atom and the oxygen of a neighbouring NO_2_ group, with a Cl1···O2^iv^ distance of 3.111 (3) Å (symmetry code as in Fig. 2). The molecular packing (Fig. 3) is further stabilized by an intermolecular C–H···π interactions between the methylene H atom of benzyloxy substituent and the 4-chlorophenyl ring of an adjacent molecule, with a C21–H21···Cg1^iii^ separation of 2.75 Å (Table 1, Cg1 is the centroid of C1-C6 benzene ring).

### Experimental

A mixture of D-glucose derived nitrone (0.5 mmol) and β-nitrostyrene (0.5 mmol) was refluxed in dry toluene (10 ml) until completion of the reaction as evidenced by TLC analysis. The solvent was evaporated under reduced pressure. The crude was purified by column chromatography on silica gel (Merck, 100-200 mesh, ethylacetate-petroleum ether (10 : 90) to afford pure isoxazolidine. Single crystals of the title compound suitable for X-ray diffraction was recrystallized from ethanol.

### Refinement

All H atoms were positioned geometrically, with C–H = 0.93-0.98 Å and constrained to ride on their parent atoms, with *U*_iso_(H) = *xU*_eq_(C, N), where *x* = 1.5 for methyl H and *x* = 1.2 for all H atoms.

### Crystal data

**Table:**

C_29_H_29_ClN_2_O_7_	*F*(000) = 1160
*M**_r_* = 552.99	*D*_x_ = 1.312 Mg m^−^^3^
Orthorhombic, *P*2_1_2_1_2_1_	Mo *K*α radiation, λ = 0.71073 Å
Hall symbol: P 2ac 2ab	Cell parameters from 3652 reflections
*a* = 12.7862 (5) Å	θ = 2.5–25°
*b* = 13.0160 (5) Å	µ = 0.19 mm^−^^1^
*c* = 16.8232 (6) Å	*T* = 293 K
*V* = 2799.80 (18) Å^3^	Needle, colourless
*Z* = 4	0.3 × 0.2 × 0.2 mm

### Data collection

**Table:**

Bruker Kappa APEXII CCD diffractometer	4764 independent reflections
Radiation source: fine-focus sealed tube	3446 reflections with *I* > 2σ(*I*)
graphite	*R*_int_ = 0.034
ω and φ scans	θ_max_ = 25.0°, θ_min_ = 2.9°
Absorption correction: multi-scan (*SADABS*; Bruker 2004)	*h* = −15→15
*T*_min_ = 0.926, *T*_max_ = 0.964	*k* = −13→14
17056 measured reflections	*l* = −16→20

### Refinement

**Table:**

Refinement on *F*^2^	Hydrogen site location: difference Fourier map
Least-squares matrix: full	H-atom parameters constrained
*R*[*F*^2^ > 2σ(*F*^2^)] = 0.037	*w* = 1/[σ^2^(*F*_o_^2^) + (0.0403*P*)^2^ + 0.2549*P*] where *P* = (*F*_o_^2^ + 2*F*_c_^2^)/3
*wR*(*F*^2^) = 0.088	(Δ/σ)_max_ < 0.001
*S* = 1.02	Δρ_max_ = 0.15 e Å^−^^3^
4764 reflections	Δρ_min_ = −0.13 e Å^−^^3^
355 parameters	Extinction correction: *SHELXL97* (Sheldrick, 2008), Fc^*^=kFc[1+0.001xFc^2^λ^3^/sin(2θ)]^-1/4^
1 restraint	Extinction coefficient: 0.0045 (6)
Primary atom site location: structure-invariant direct methods	Absolute structure: Flack (1983), 1975 Friedel pairs
Secondary atom site location: difference Fourier map	Flack parameter: −0.05 (8)

### Special details

**Table:**

Geometry. All esds (except the esd in the dihedral angle between two l.s. planes) are estimated using the full covariance matrix. The cell esds are taken into account individually in the estimation of esds in distances, angles and torsion angles; correlations between esds in cell parameters are only used when they are defined by crystal symmetry. An approximate (isotropic) treatment of cell esds is used for estimating esds involving l.s. planes.
Refinement. Refinement of F^2^ against ALL reflections. The weighted R-factor wR and goodness of fit S are based on F^2^, conventional R-factors R are based on F, with F set to zero for negative F^2^. The threshold expression of F^2^ > 2sigma(F^2^) is used only for calculating R-factors(gt) etc. and is not relevant to the choice of reflections for refinement. R-factors based on F^2^ are statistically about twice as large as those based on F, and R- factors based on ALL data will be even larger.

### Fractional atomic coordinates and isotropic or equivalent isotropic displacement parameters (Å^2^)

**Table:**

	*x*	*y*	*z*	*U*_iso_*/*U*_eq_	
C1	0.33984 (19)	0.6866 (2)	0.65294 (16)	0.0602 (7)	
H1	0.3415	0.6459	0.6983	0.072*	
C2	0.2814 (2)	0.7761 (2)	0.65257 (18)	0.0692 (7)	
H2	0.2457	0.7964	0.6981	0.083*	
C3	0.2765 (2)	0.8340 (2)	0.5857 (2)	0.0673 (7)	
C4	0.3326 (2)	0.8082 (2)	0.5200 (2)	0.0760 (9)	
H4	0.3301	0.8493	0.4749	0.091*	
C5	0.3933 (2)	0.7205 (2)	0.52082 (18)	0.0691 (8)	
H5	0.4332	0.7039	0.4764	0.083*	
C6	0.39575 (17)	0.65738 (18)	0.58632 (15)	0.0471 (6)	
C7	0.45334 (17)	0.55719 (17)	0.58177 (14)	0.0455 (6)	
H7	0.5181	0.5658	0.5513	0.055*	
C8	0.38927 (17)	0.46762 (17)	0.54776 (13)	0.0427 (6)	
H8	0.3143	0.4808	0.5544	0.051*	
C9	0.42248 (17)	0.37460 (18)	0.59803 (14)	0.0434 (6)	
H9	0.4536	0.3214	0.5643	0.052*	
C10	0.33056 (15)	0.33112 (18)	0.64465 (14)	0.0434 (6)	
H10	0.3092	0.3802	0.6857	0.052*	
C11	0.34793 (16)	0.22621 (18)	0.68184 (14)	0.0448 (6)	
H11	0.3831	0.2310	0.7335	0.054*	
C12	0.23650 (18)	0.18767 (19)	0.68975 (14)	0.0474 (6)	
H12	0.2314	0.1126	0.6886	0.057*	
C13	0.17952 (16)	0.23875 (19)	0.62140 (14)	0.0460 (6)	
H13	0.1606	0.1884	0.5805	0.055*	
C14	0.08681 (19)	0.2635 (2)	0.73843 (15)	0.0582 (7)	
C15	0.0626 (3)	0.3618 (3)	0.7812 (2)	0.1063 (12)	
H15A	0.1132	0.4131	0.7668	0.159*	
H15B	0.0653	0.3503	0.8375	0.159*	
H15C	−0.0060	0.3849	0.7666	0.159*	
C16	0.0104 (2)	0.1788 (3)	0.7552 (2)	0.0866 (10)	
H16A	−0.0575	0.1981	0.7359	0.130*	
H16B	0.0069	0.1669	0.8114	0.130*	
H16C	0.0328	0.1171	0.7289	0.130*	
C17	0.4468 (2)	0.0741 (2)	0.66159 (16)	0.0585 (7)	
H17A	0.3903	0.0319	0.6819	0.070*	
H17B	0.4917	0.0925	0.7058	0.070*	
C18	0.50869 (17)	0.01390 (19)	0.60141 (15)	0.0493 (6)	
C19	0.5602 (2)	−0.0733 (2)	0.62669 (19)	0.0713 (8)	
H19	0.5570	−0.0928	0.6798	0.086*	
C20	0.6160 (2)	−0.1312 (3)	0.5736 (2)	0.0875 (10)	
H20	0.6507	−0.1899	0.5910	0.105*	
C21	0.6215 (2)	−0.1038 (3)	0.4954 (2)	0.0811 (9)	
H21	0.6591	−0.1439	0.4597	0.097*	
C22	0.5717 (2)	−0.0176 (2)	0.46998 (17)	0.0713 (8)	
H22	0.5757	0.0018	0.4169	0.086*	
C23	0.5151 (2)	0.0412 (2)	0.52292 (16)	0.0608 (7)	
H23	0.4809	0.1000	0.5052	0.073*	
C24	0.60915 (17)	0.3964 (2)	0.63870 (14)	0.0489 (6)	
C25	0.63995 (19)	0.2974 (2)	0.62056 (17)	0.0639 (8)	
H25	0.5905	0.2457	0.6140	0.077*	
C26	0.7453 (2)	0.2761 (3)	0.61224 (17)	0.0790 (9)	
H26	0.7663	0.2100	0.5988	0.095*	
C27	0.8194 (2)	0.3512 (3)	0.62345 (18)	0.0847 (11)	
H27	0.8901	0.3359	0.6187	0.102*	
C28	0.7876 (2)	0.4485 (3)	0.64162 (19)	0.0805 (10)	
H28	0.8373	0.4999	0.6485	0.097*	
C29	0.68311 (17)	0.4720 (2)	0.64995 (16)	0.0618 (7)	
H29	0.6626	0.5384	0.6631	0.074*	
N1	0.50029 (14)	0.41439 (15)	0.65469 (12)	0.0476 (5)	
N2	0.4148 (2)	0.45251 (17)	0.46170 (13)	0.0574 (6)	
O1	0.47679 (12)	0.52172 (12)	0.66073 (9)	0.0517 (4)	
O2	0.4993 (2)	0.4215 (2)	0.44423 (13)	0.0993 (8)	
O3	0.3483 (2)	0.4733 (2)	0.41370 (13)	0.1086 (8)	
O4	0.24623 (11)	0.31531 (12)	0.59000 (9)	0.0503 (4)	
O5	0.19043 (12)	0.23172 (14)	0.75864 (9)	0.0588 (5)	
O6	0.08979 (12)	0.28308 (14)	0.65527 (10)	0.0613 (5)	
O7	0.40491 (11)	0.16447 (12)	0.62710 (9)	0.0478 (4)	
Cl1	0.19572 (8)	0.94167 (6)	0.58228 (7)	0.1059 (3)	

### Atomic displacement parameters (Å^2^)

**Table:**

	*U*^11^	*U*^22^	*U*^33^	*U*^12^	*U*^13^	*U*^23^
C1	0.0647 (16)	0.0588 (18)	0.0572 (16)	0.0114 (14)	−0.0014 (14)	−0.0080 (14)
C2	0.0688 (17)	0.070 (2)	0.0692 (16)	0.0167 (16)	−0.0049 (15)	−0.0232 (13)
C3	0.0706 (18)	0.0432 (17)	0.0883 (19)	0.0018 (14)	−0.0139 (17)	−0.0167 (13)
C4	0.099 (2)	0.0429 (18)	0.086 (2)	−0.0020 (17)	−0.0028 (19)	0.0106 (16)
C5	0.088 (2)	0.0506 (18)	0.0687 (19)	−0.0036 (16)	0.0181 (16)	−0.0007 (15)
C6	0.0449 (13)	0.0404 (15)	0.0559 (15)	−0.0077 (11)	0.0023 (12)	−0.0126 (13)
C7	0.0447 (13)	0.0421 (15)	0.0497 (15)	−0.0055 (11)	0.0041 (11)	−0.0071 (12)
C8	0.0416 (12)	0.0425 (14)	0.0439 (14)	−0.0004 (11)	0.0024 (10)	−0.0035 (11)
C9	0.0385 (12)	0.0433 (15)	0.0485 (14)	0.0025 (10)	−0.0014 (11)	−0.0027 (12)
C10	0.0388 (12)	0.0432 (15)	0.0482 (14)	0.0006 (11)	−0.0034 (10)	−0.0004 (11)
C11	0.0452 (13)	0.0443 (15)	0.0450 (13)	0.0031 (11)	−0.0028 (11)	0.0019 (12)
C12	0.0530 (14)	0.0414 (15)	0.0479 (14)	0.0007 (12)	0.0052 (11)	0.0001 (12)
C13	0.0439 (12)	0.0471 (15)	0.0471 (13)	−0.0059 (12)	0.0000 (10)	−0.0003 (12)
C14	0.0540 (15)	0.0662 (19)	0.0544 (16)	0.0074 (14)	0.0049 (13)	−0.0018 (14)
C15	0.120 (3)	0.101 (3)	0.099 (3)	0.031 (2)	0.007 (2)	−0.033 (2)
C16	0.0640 (18)	0.104 (3)	0.091 (2)	−0.0119 (19)	0.0126 (16)	0.026 (2)
C17	0.0702 (17)	0.0496 (17)	0.0556 (16)	0.0215 (14)	−0.0020 (13)	0.0105 (13)
C18	0.0447 (13)	0.0441 (16)	0.0592 (17)	0.0053 (12)	0.0004 (11)	0.0064 (13)
C19	0.0730 (18)	0.065 (2)	0.076 (2)	0.0201 (17)	0.0068 (15)	0.0156 (16)
C20	0.078 (2)	0.069 (2)	0.115 (3)	0.0330 (17)	0.018 (2)	0.013 (2)
C21	0.0659 (19)	0.065 (2)	0.112 (3)	0.0099 (17)	0.0286 (18)	−0.014 (2)
C22	0.0799 (19)	0.069 (2)	0.0653 (19)	−0.0030 (18)	0.0187 (16)	−0.0046 (16)
C23	0.0703 (17)	0.0506 (17)	0.0615 (18)	0.0124 (14)	0.0001 (14)	0.0040 (14)
C24	0.0386 (13)	0.0631 (19)	0.0451 (15)	0.0009 (13)	−0.0026 (11)	0.0005 (12)
C25	0.0474 (14)	0.070 (2)	0.0742 (18)	0.0103 (14)	−0.0031 (13)	−0.0081 (15)
C26	0.0579 (17)	0.100 (3)	0.080 (2)	0.0289 (19)	0.0031 (15)	−0.0037 (19)
C27	0.0411 (16)	0.138 (3)	0.075 (2)	0.015 (2)	0.0063 (14)	0.023 (2)
C28	0.0423 (15)	0.117 (3)	0.082 (2)	−0.0142 (18)	−0.0024 (14)	0.024 (2)
C29	0.0479 (14)	0.0722 (19)	0.0652 (17)	−0.0065 (14)	−0.0031 (12)	0.0012 (15)
N1	0.0396 (11)	0.0478 (13)	0.0553 (12)	0.0014 (9)	−0.0010 (9)	−0.0058 (10)
N2	0.0776 (15)	0.0449 (13)	0.0497 (14)	−0.0107 (12)	−0.0008 (13)	−0.0059 (11)
O1	0.0526 (9)	0.0512 (11)	0.0513 (11)	0.0034 (8)	−0.0048 (7)	−0.0116 (9)
O2	0.1055 (18)	0.123 (2)	0.0690 (14)	0.0167 (16)	0.0285 (13)	−0.0203 (13)
O3	0.136 (2)	0.125 (2)	0.0645 (14)	0.0038 (17)	−0.0251 (14)	0.0137 (14)
O4	0.0404 (8)	0.0540 (11)	0.0565 (10)	−0.0071 (8)	−0.0082 (7)	0.0133 (8)
O5	0.0541 (10)	0.0757 (13)	0.0465 (10)	0.0024 (9)	0.0008 (8)	0.0010 (9)
O6	0.0485 (10)	0.0762 (13)	0.0591 (11)	0.0070 (9)	0.0053 (8)	0.0097 (10)
O7	0.0547 (9)	0.0422 (10)	0.0467 (9)	0.0116 (8)	−0.0005 (7)	0.0056 (8)
Cl1	0.1104 (7)	0.0618 (5)	0.1454 (9)	0.0290 (5)	−0.0323 (6)	−0.0224 (6)

### Geometric parameters (Å, °)

**Table:**

C1—C6	1.383 (3)	C15—H15A	0.9600
C1—C2	1.383 (4)	C15—H15B	0.9600
C1—H1	0.9300	C15—H15C	0.9600
C2—C3	1.355 (4)	C16—H16A	0.9600
C2—H2	0.9300	C16—H16B	0.9600
C3—C4	1.360 (4)	C16—H16C	0.9600
C3—Cl1	1.742 (3)	C17—O7	1.417 (3)
C4—C5	1.380 (4)	C17—C18	1.505 (3)
C4—H4	0.9300	C17—H17A	0.9700
C5—C6	1.375 (4)	C17—H17B	0.9700
C5—H5	0.9300	C18—C23	1.370 (3)
C6—C7	1.500 (3)	C18—C19	1.380 (3)
C7—O1	1.438 (3)	C19—C20	1.369 (4)
C7—C8	1.535 (3)	C19—H19	0.9300
C7—H7	0.9800	C20—C21	1.364 (5)
C8—N2	1.497 (3)	C20—H20	0.9300
C8—C9	1.537 (3)	C21—C22	1.360 (4)
C8—H8	0.9800	C21—H21	0.9300
C9—N1	1.472 (3)	C22—C23	1.380 (4)
C9—C10	1.522 (3)	C22—H22	0.9300
C9—H9	0.9800	C23—H23	0.9300
C10—O4	1.432 (3)	C24—C29	1.378 (3)
C10—C11	1.518 (3)	C24—C25	1.382 (4)
C10—H10	0.9800	C24—N1	1.437 (3)
C11—O7	1.423 (3)	C25—C26	1.382 (4)
C11—C12	1.516 (3)	C25—H25	0.9300
C11—H11	0.9800	C26—C27	1.374 (4)
C12—O5	1.421 (3)	C26—H26	0.9300
C12—C13	1.515 (3)	C27—C28	1.366 (5)
C12—H12	0.9800	C27—H27	0.9300
C13—O6	1.405 (3)	C28—C29	1.377 (4)
C13—O4	1.414 (3)	C28—H28	0.9300
C13—H13	0.9800	C29—H29	0.9300
C14—O6	1.423 (3)	N1—O1	1.433 (2)
C14—O5	1.429 (3)	N2—O2	1.191 (3)
C14—C15	1.500 (4)	N2—O3	1.203 (3)
C14—C16	1.500 (4)	Cl1—O2^i^	3.111 (3)
			
C6—C1—C2	120.5 (3)	C14—C15—H15A	109.5
C6—C1—H1	119.8	C14—C15—H15B	109.5
C2—C1—H1	119.8	H15A—C15—H15B	109.5
C3—C2—C1	119.8 (3)	C14—C15—H15C	109.5
C3—C2—H2	120.1	H15A—C15—H15C	109.5
C1—C2—H2	120.1	H15B—C15—H15C	109.5
C2—C3—C4	120.8 (3)	C14—C16—H16A	109.5
C2—C3—Cl1	120.2 (2)	C14—C16—H16B	109.5
C4—C3—Cl1	119.0 (3)	H16A—C16—H16B	109.5
C3—C4—C5	119.6 (3)	C14—C16—H16C	109.5
C3—C4—H4	120.2	H16A—C16—H16C	109.5
C5—C4—H4	120.2	H16B—C16—H16C	109.5
C6—C5—C4	121.0 (3)	O7—C17—C18	110.8 (2)
C6—C5—H5	119.5	O7—C17—H17A	109.5
C4—C5—H5	119.5	C18—C17—H17A	109.5
C5—C6—C1	118.3 (2)	O7—C17—H17B	109.5
C5—C6—C7	119.3 (2)	C18—C17—H17B	109.5
C1—C6—C7	122.3 (2)	H17A—C17—H17B	108.1
O1—C7—C6	109.53 (18)	C23—C18—C19	118.9 (2)
O1—C7—C8	102.22 (18)	C23—C18—C17	123.0 (2)
C6—C7—C8	114.67 (18)	C19—C18—C17	118.2 (2)
O1—C7—H7	110.0	C20—C19—C18	120.0 (3)
C6—C7—H7	110.0	C20—C19—H19	120.0
C8—C7—H7	110.0	C18—C19—H19	120.0
N2—C8—C7	110.11 (18)	C21—C20—C19	120.8 (3)
N2—C8—C9	111.63 (19)	C21—C20—H20	119.6
C7—C8—C9	104.23 (17)	C19—C20—H20	119.6
N2—C8—H8	110.2	C22—C21—C20	119.7 (3)
C7—C8—H8	110.2	C22—C21—H21	120.2
C9—C8—H8	110.2	C20—C21—H21	120.2
N1—C9—C10	108.61 (18)	C21—C22—C23	120.0 (3)
N1—C9—C8	105.42 (17)	C21—C22—H22	120.0
C10—C9—C8	111.29 (18)	C23—C22—H22	120.0
N1—C9—H9	110.5	C18—C23—C22	120.6 (3)
C10—C9—H9	110.5	C18—C23—H23	119.7
C8—C9—H9	110.5	C22—C23—H23	119.7
O4—C10—C11	104.21 (17)	C29—C24—C25	120.1 (2)
O4—C10—C9	107.70 (18)	C29—C24—N1	121.5 (2)
C11—C10—C9	115.70 (18)	C25—C24—N1	118.0 (2)
O4—C10—H10	109.7	C24—C25—C26	119.1 (3)
C11—C10—H10	109.7	C24—C25—H25	120.4
C9—C10—H10	109.7	C26—C25—H25	120.4
O7—C11—C12	110.55 (18)	C27—C26—C25	121.1 (3)
O7—C11—C10	108.43 (18)	C27—C26—H26	119.5
C12—C11—C10	101.32 (17)	C25—C26—H26	119.5
O7—C11—H11	112.0	C28—C27—C26	119.0 (3)
C12—C11—H11	112.0	C28—C27—H27	120.5
C10—C11—H11	112.0	C26—C27—H27	120.5
O5—C12—C13	104.04 (18)	C27—C28—C29	121.2 (3)
O5—C12—C11	109.12 (19)	C27—C28—H28	119.4
C13—C12—C11	103.89 (18)	C29—C28—H28	119.4
O5—C12—H12	113.0	C28—C29—C24	119.5 (3)
C13—C12—H12	113.0	C28—C29—H29	120.2
C11—C12—H12	113.0	C24—C29—H29	120.2
O6—C13—O4	110.77 (19)	O1—N1—C24	112.06 (18)
O6—C13—C12	105.37 (18)	O1—N1—C9	104.32 (16)
O4—C13—C12	107.62 (17)	C24—N1—C9	118.42 (18)
O6—C13—H13	111.0	O2—N2—O3	123.5 (3)
O4—C13—H13	111.0	O2—N2—C8	118.8 (2)
C12—C13—H13	111.0	O3—N2—C8	117.8 (2)
O6—C14—O5	105.13 (18)	N1—O1—C7	106.94 (16)
O6—C14—C15	108.9 (2)	C13—O4—C10	108.40 (16)
O5—C14—C15	108.9 (2)	C12—O5—C14	107.88 (17)
O6—C14—C16	109.5 (2)	C13—O6—C14	110.31 (18)
O5—C14—C16	110.3 (2)	C17—O7—C11	113.41 (18)
C15—C14—C16	113.8 (3)	C3—Cl1—O2^i^	160.74 (11)
			
C6—C1—C2—C3	1.9 (4)	C17—C18—C23—C22	−179.1 (3)
C1—C2—C3—C4	−3.4 (4)	C21—C22—C23—C18	0.3 (4)
C1—C2—C3—Cl1	175.3 (2)	C29—C24—C25—C26	−1.4 (4)
C2—C3—C4—C5	1.6 (4)	N1—C24—C25—C26	−174.0 (2)
Cl1—C3—C4—C5	−177.2 (2)	C24—C25—C26—C27	1.5 (4)
C3—C4—C5—C6	1.8 (4)	C25—C26—C27—C28	−1.2 (5)
C4—C5—C6—C1	−3.2 (4)	C26—C27—C28—C29	1.0 (5)
C4—C5—C6—C7	173.4 (2)	C27—C28—C29—C24	−0.9 (4)
C2—C1—C6—C5	1.4 (4)	C25—C24—C29—C28	1.1 (4)
C2—C1—C6—C7	−175.1 (2)	N1—C24—C29—C28	173.5 (2)
C5—C6—C7—O1	160.2 (2)	C29—C24—N1—O1	16.3 (3)
C1—C6—C7—O1	−23.3 (3)	C25—C24—N1—O1	−171.2 (2)
C5—C6—C7—C8	−85.6 (3)	C29—C24—N1—C9	137.8 (2)
C1—C6—C7—C8	90.9 (3)	C25—C24—N1—C9	−49.7 (3)
O1—C7—C8—N2	−143.01 (19)	C10—C9—N1—O1	−95.32 (19)
C6—C7—C8—N2	98.6 (2)	C8—C9—N1—O1	24.0 (2)
O1—C7—C8—C9	−23.2 (2)	C10—C9—N1—C24	139.3 (2)
C6—C7—C8—C9	−141.6 (2)	C8—C9—N1—C24	−101.3 (2)
N2—C8—C9—N1	118.5 (2)	C7—C8—N2—O2	68.6 (3)
C7—C8—C9—N1	−0.3 (2)	C9—C8—N2—O2	−46.6 (3)
N2—C8—C9—C10	−124.0 (2)	C7—C8—N2—O3	−110.8 (2)
C7—C8—C9—C10	117.22 (19)	C9—C8—N2—O3	133.9 (2)
N1—C9—C10—O4	166.62 (17)	C24—N1—O1—C7	88.2 (2)
C8—C9—C10—O4	51.0 (2)	C9—N1—O1—C7	−41.12 (19)
N1—C9—C10—C11	−77.3 (2)	C6—C7—O1—N1	161.96 (16)
C8—C9—C10—C11	167.08 (18)	C8—C7—O1—N1	39.96 (19)
O4—C10—C11—O7	78.2 (2)	O6—C13—O4—C10	101.9 (2)
C9—C10—C11—O7	−39.9 (2)	C12—C13—O4—C10	−12.8 (2)
O4—C10—C11—C12	−38.2 (2)	C11—C10—O4—C13	32.4 (2)
C9—C10—C11—C12	−156.24 (19)	C9—C10—O4—C13	155.77 (18)
O7—C11—C12—O5	164.63 (18)	C13—C12—O5—C14	26.3 (2)
C10—C11—C12—O5	−80.6 (2)	C11—C12—O5—C14	136.67 (19)
O7—C11—C12—C13	−84.9 (2)	O6—C14—O5—C12	−26.7 (3)
C10—C11—C12—C13	29.9 (2)	C15—C14—O5—C12	−143.2 (2)
O5—C12—C13—O6	−15.9 (2)	C16—C14—O5—C12	91.3 (2)
C11—C12—C13—O6	−130.13 (19)	O4—C13—O6—C14	−116.2 (2)
O5—C12—C13—O4	102.3 (2)	C12—C13—O6—C14	−0.1 (3)
C11—C12—C13—O4	−11.9 (2)	O5—C14—O6—C13	16.1 (3)
O7—C17—C18—C23	−4.6 (3)	C15—C14—O6—C13	132.6 (2)
O7—C17—C18—C19	176.1 (2)	C16—C14—O6—C13	−102.4 (2)
C23—C18—C19—C20	−0.3 (4)	C18—C17—O7—C11	−178.33 (18)
C17—C18—C19—C20	179.0 (3)	C12—C11—O7—C17	−83.7 (2)
C18—C19—C20—C21	−0.1 (5)	C10—C11—O7—C17	166.09 (18)
C19—C20—C21—C22	0.6 (5)	C2—C3—Cl1—O2^i^	−90.1 (5)
C20—C21—C22—C23	−0.7 (5)	C4—C3—Cl1—O2^i^	88.7 (5)
C19—C18—C23—C22	0.2 (4)		

Symmetry codes: (i) *x*−1/2, −*y*+3/2, −*z*+1.

### Hydrogen-bond geometry (Å, °)

**Table:**

*D*—H···*A*	*D*—H	H···*A*	*D*···*A*	*D*—H···*A*
C13—H13···O2^ii^	0.98	2.54	3.298 (3)	134
C17—H17B···O1^iii^	0.97	2.46	3.218 (3)	135
C21—H21···Cg1^ii^	0.93	2.75	3.598 (1)	152

Symmetry codes: (ii) *x*−1/2, −*y*+1/2, −*z*+1; (iii) −*x*+1, *y*−1/2, −*z*+3/2.
